# High-Dimensional Phase Space Reconstruction with a Convolutional Neural Network for Structural Health Monitoring

**DOI:** 10.3390/s21103514

**Published:** 2021-05-18

**Authors:** Yen-Lin Chen, Yuan Chiang, Pei-Hsin Chiu, I-Chen Huang, Yu-Bai Xiao, Shu-Wei Chang, Chang-Wei Huang

**Affiliations:** 1 Department of Civil Engineering, National Taiwan University, Taipei City 10617, Taiwan; b05501056@ntu.edu.tw (Y.-L.C.); r08521203@ntu.edu.tw (Y.C.); r07521219@ntu.edu.tw (P.-H.C.); r07521223@ntu.edu.tw (Y.-B.X.); 2 Department of Civil Engineering, Chung Yuan Christian University, Taoyuan City 320314, Taiwan; yichuang@tylin.com.tw

**Keywords:** convolutional neural network (CNN), structural health monitoring (SHM), Takens’ embedding theorem, attractor reconstruction

## Abstract

In order to accurately diagnose the health of high-order statically indeterminate structures, most existing structural health monitoring (SHM) methods require multiple sensors to collect enough information. However, comprehensive data collection from multiple sensors for high degree-of-freedom structures is not typically available in practice. We propose a method that reconciles the two seemingly conflicting difficulties. Takens’ embedding theorem is used to augment the dimensions of data collected from a single sensor. Taking advantage of the success of machine learning in image classification, high-dimensional reconstructed attractors were converted into images and fed into a convolutional neural network (CNN). Attractor classification was performed for 10 damage cases of a 3-story shear frame structure. Numerical results show that the inherently high dimension of the CNN model allows the handling of higher dimensional data. Information on both the level and the location of damage was successfully embedded. The same methodology will allow the extraction of data with unsupervised CNN classification to be consistent with real use cases.

## 1. Introduction

Structures suffer from varying levels of damage during their lifespan due to aging, external forces, and environmental changes. The implementation of damage detection for structures, known as structural health monitoring (SHM), is key for both preventing unexpected failures and optimizing the maintenance of existing structures [1,2,3]. As a result, SHM is indispensable for the sustainability of structures and therefore attracts extensive attention in both the industry and academia. The goals of SHM can be classified into four categories [4]: (1) to determine whether the structure is in a healthy state, (2) to identify the location of damage, (3) to quantify the level of damage, and (4) to estimate the remaining service life of the structure. 

One common approach to SHM is the vibration-based method, where the response to an excitation is analyzed [4,5,6,7,8,9] The underlying assumption is that the change in the level of damage can be observed as a change in the structure’s responses, such as natural frequencies and damping ratios. It follows that the primary task for vibration-based approaches is to quantify the difference between responses. This type of SHM is performed with the aid of various sensors, such as accelerometers, displacement transducers, and strain gauges, to measure the time-history response of the structure in question.

Vibration-based methods can be further separated into two categories: model-based and feature-based methods. Model-based methods correlate the state of the structure with a predetermined model for structural health diagnosis [10]. Some common models are the autoregressive and moving average (ARMA) model [11,12] and the Hidden Markov Model (HMM). Feature-based methods are generally performed in two steps: feature extraction and feature comparison [10]. Among these methods, modal analysis is the most common method for extracting features, such as the use of natural frequency, modal shape, and modal damping [13,14,15,16]. All these methods are based on a comparison of the global properties of the collected data. However, it has been reported that SHM based on modal analysis is insensitive to minor local damage in practice [17]. A review on the modal analysis approach can be found in [18,19].

Another property used by feature-based approaches is the attractor of the system. The advantage of attractor-based methods (categorized as feature-based methods) is in their independence from a predetermined model, since the attractor itself is the model [20]. Various metrics are used in these methods, including prediction error [21], local attractor variance [22], and continuity [23]. Each metric requires iteration over the data points in the attractor. According to Nichols [24], the number of points required to accurately describe (i.e., populate) an attractor is proportional to a power of the number of degrees-of-freedom (DOF). Not only does this imply the necessity of extensive observation, but it also implies that these methods are efficient only for a small number of DOFs. Consequently, these methods implement dimension reduction before construction of the attractor, which inevitably reduces the amount of information available. In addition to the issue of dimension reduction, these methods are proposed under the assumption that the system is deterministic—only then do attractors exist. To obtain a deterministic system, specific excitations must be prescribed. However, this is not generally applicable.

The extensibility of attractor-related vibration-based methods to stochastic systems with pullback attractors was investigated by Overbey et al. [25], where it was found that vibration-based methods are applicable to stochastic excitation, including band-limited white noise. Since the main concern in using ambient excitation as input is the signal being neither ergodic nor stationary, this finding raises the possibility of its usage. This signal is inexpensive and has the additional benefit of being available at any time. Despite the ease of application, caution is needed when employing ambient signals. As ambient excitation varies as a function of environmental properties such as temperature and humidity, the suitable technique also changes depending on the properties of the entire system [26].

Ever-increasing computational power has allowed the current mainstream machine learning techniques, in particular deep neural networks (DNNs). The major advantages of machine learning-based techniques focus on two steps: (1) feature extraction and (2) feature discrimination in the SHM process [27]. In recent years, both one- and two-dimensional convolutional neural networks (CNNs) have shown superiority over conventional methods in accuracy and efficiency, largely due to the combination of feature extraction and feature discrimination steps into a single learning block [28].

Extending the concept of comparing attractors, a useful feature of DNNs is their large number of nodes, making DNNs readily available for the large number of points required to accommodate an attractor with high dimensions. Taking into consideration the recent success and ease of implementation of CNNs, we ask the following questions: Is there a reasonable method for converting a high-dimensional attractor into a matrix, such that no information is lost? Furthermore, can a CNN be readily used to distinguish between healthy and non-healthy structures?

**Figure 1:**
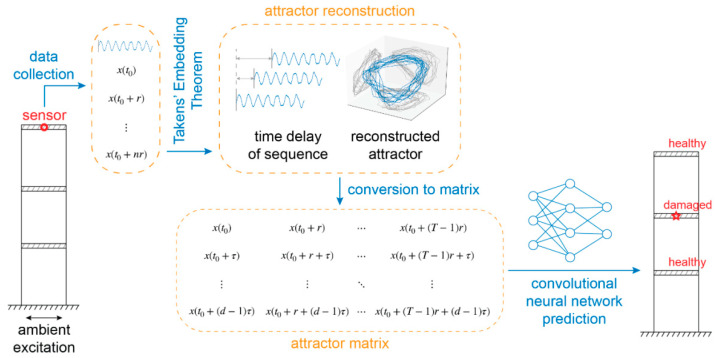
Classification scheme. Data collected from a single sensor from the structure is converted into a matrix based on Takens’ embedding theorem. The matrix is then fed into a convolutional neural network for feature extraction and classification of the underlying structure’s level of structural damage and for determining the location of damage.

To tackle these questions, Takens’ embedding theorem [29] is utilized to recover a high-dimensional attractor representative of the system from data collected by a single sensor in the structure. The method converts this attractor into a matrix/image. The converted product is then fed into a CNN for the diagnosis of the health of the structure (Figure 1). Our results demonstrate that reconstructed attractors of different damage cases can be discriminated by the CNN model. The proposed combinatorial numerical framework therefore provides a novel and reliable method for SHM and furthers the progress toward future SHM schemes that lack prescribed damage data.

## 2. Materials and Methods

The proposed SHM method is based on two ingredients: A high-dimensional attractor from a single sensor is first reconstructed on the basis of Takens’ embedding theorem. Then the reconstructed attractor is converted into two-dimensional images and is identified by the CNN to obtain the features of attractors. Details of each step are discussed in this section. Moreover, to test the proposed computational framework, a three-story shear frame with varying damage magnitude and location was constructed to generate the time-history responses.

### 2.1. Attractor Reconstruction

In SHM, a structure can be seen as a function that takes the excitation (either ambient or prescribed) as input and outputs a response that can be picked up by sensors, typically in the form of displacement or acceleration. This dynamic system can be completely described by its phase space representation of m dimensions, where m is the number of DOFs of the system. For deterministic systems, its trajectory in the phase space asymptotically approaches a certain system-dependent manifold: the attractor. However, obtaining the trajectory in the phase space requires m sensors, one for each DOF. This is improbable in most cases and thus some alternative method for obtaining the attractor of the system is necessary. The goal of such a method is to construct an attractor in an alternative space (different from that constructed from m independent sensors), such that the new attractor is completely unfolded (i.e., no overlapping points exist on the attractor).

The time delay method is the most common approach for attractor reconstruction. At the core of this method is Takens’ embedding theorem, which states that the time-history response collected from just one sensor is sufficient for the reconstruction. Interested readers are referred to the original study [29], as well as an alternative proof [30].

**Figure 2:**
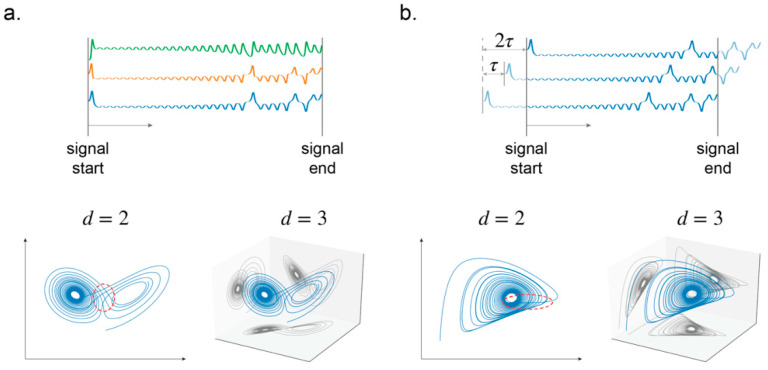
The effect of incrementing dimension on the reconstructed attractor. The two- and three-dimensional manifold of the Lorenz attractor. (**a**) Without time delay, where d=2 can be seen as a projection of the three-dimensional signal on a 2D plane. (**b**) With time delay. The overlapping trajectory is marked on the two-dimensional plot.

To reconstruct the attractor using a single sensor (observation function), the dimension of the space must be expanded. The necessity of such an expansion in dimension is illustrated in Figure 2. Time delay vectors $\vec{y}$ are created for this purpose, given by

(1) $$\vec{y}\left(t\right)=\langle x\left(t\right),x\left(t+\tau \right),x\left(t+2\tau \right),\cdots ,x\left(t+\left(d-1\right)\tau \right),$$

where x(t) denotes the observation at time t, τ is the delay time, and d is the embedding dimension. Here, both τ and d are yet to be chosen. A trajectory approximating the attractor is constructed by plotting the sequence

(2) $$\left\{\vec{y}\left(t_{0}\right),\vec{y}\left(t_{0}+r\right),\vec{y}\left(t_{0}+2r\right),\cdots ,\vec{y}\left(t_{0}+\left(T-1\right)r\right)\right\},$$

in the d-dimensional space, also known as the reconstruction space. Here, $t_{0}$ is the time of the first observation in the whole sequence, r is the sample interval of each vector, chosen to be sufficiently small so as to preserve the local pattern of the trajectory, and T determines the length of the reconstructed trajectory, which has to be sufficiently large for the geometry of the attractor to be observable. Both the sample interval r and the trajectory length T are chosen by taking the characteristic time of the system into consideration. Hence, an understanding of the governing physics is necessary.

In practice, τ is usually taken as a multiple of r, such that most observations between Equations (1) and (2) are shared to reduce the number of observations required. It must also take into consideration the sample rate of the sensor, since no sensor records in continuous time. The ideal choice of τ is one that maximizes the information gained from the time delay vector. Practical choices for τ are the first minimum of the average mutual information (AMI) function or the first zero of autocorrelation. An illustration of the reconstruction is given in Figure 2b.

Unlike the delay time that has an optimal value, there is only a lower bound for the embedding dimension d. The goal is to choose a sufficiently large d, such that the attractor is completely unfolded in the reconstruction space. In Whitney’s embedding theorem, 2m+1 dimensions suffice, where m is the number of DOFs of the system. However, since the actual number of DOFs of the system is unknown in practice, d has to be chosen experimentally, for instance, by making use of the false nearest neighbor (FNN) method. The concept of FNN is straightforward: true neighbors on an attractor remain close even if the attractor is reconstructed in a higher dimension, and those that become separated in a higher dimension only seem close due to projection onto a lower dimension. Using this concept, this method constructs the attractor in spaces of increasing dimension, and the iteration terminates when all nearest neighbors remain close after the dimension increment. The final dimension thus gives the minimum value for unfolding the attractor and is taken as the embedding dimension d.

### 2.2. Attractor Matrix for SHM

With the above theory, a method is proposed for converting the time-history response into a matrix. The goal is to preserve the information on attractors; τ, d, and T have to be determined beforehand. Differing from the methods given above, the value of both d and T can be chosen without significant consideration of computational expense since the matrix is to be processed with machine learning techniques. That is, values for d and T can be chosen such that the practitioner is confident in their magnitude. With these values determined, the matrix is constructed as follows.

**Figure 3:**
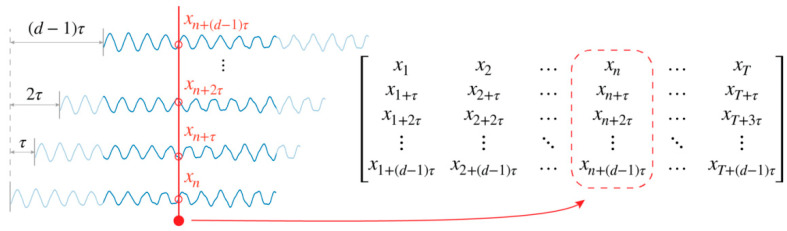
Conversion from sequence to attractor matrix. A single signal is delayed by τ, thus creating a new signal. The d signals are gathered and put into a matrix to form the attractor matrix.

Given a system, first define an observation function that returns a value x(t) when called at time t. Collect the sequence consisting of T time delay vectors as in Equation (2) by the observation function. Taking each time delay vector in the sequence as single-column vectors, a matrix in the ${\mathbb{R}}^{d\times T}$ space is formed:

(3) $$\left[\begin{array}{ccccc} x\left(t_{0}\right) & x\left(t_{0}+r\right) & x\left(t_{0}+2r\right) & \cdots & x\left(t_{0}+\left(T-1\right)r\right)\\ x\left(t_{0}+\tau \right) & x\left(t_{0}+r+\tau \right) & x\left(t_{0}+2r+\tau \right) & \cdots & x\left(t_{0}+\left(T-1\right)r+\tau \right)\\ \vdots & \vdots & \vdots & \ddots & \vdots \\ x\left(t_{0}+\left(d-1\right)\tau \right) & x\left(t_{0}+r+\left(d-1\right)\tau \right) & x\left(t_{0}+2r+\left(d-1\right)\tau \right) & \cdots & x\left(t_{0}+\left(T-1\right)r+\left(d-1\right)\tau \right) \end{array}\right],$$

where the notation follows Section 2.1. This matrix is referred to here as the “attractor matrix”. The scheme of the conversion is illustrated in Figure 3.

To further present the matrix as an image, the values in the matrix are shifted and scaled to 0–255. Each element of the matrix then represents a pixel in a grayscale image. For a set with multiple attractor matrices, the information on relative amplitude will be lost if the shifting and scaling is done independently on each individual matrix. When this is not the desired effect, the shifting and scaling should be done with respect to the entire dataset. Instead of finding the maximum and minimum values with respect to each matrix for shifting and scaling, these two values should be found with respect to the entire dataset, with scaling and shifting done globally as well. This preserves the information on relative magnitude.

### 2.3. Numerical Model

The phase space reconstruction outlined in Section 2.1 is designed for deterministic systems with a deterministic attractor. However, as validated experimentally by Overbey et al. [25], the method can be applied without issue to certain stochastic systems, including structures with band-limited white noise as excitation.

Given that the goal of this work is to establish a method for converting reconstructed attractors into matrices/images without losing details on the level of damage, the purpose of this demonstration is to show that the information regarding the health of the structure is preserved and is readily extractible with CNN. It also aims to show that information regarding the location of damage is included in the matrix. To this end, the conversion–CNN workflow is applied to a simulated system. A simple structure is simulated with varying damage magnitude and location, and the acceleration response to white noise excitation is converted to images using the proposed conversion. These images are then used to train a CNN model under the image classification scheme. This property is considered as properly embedded and readily extractible in the image/matrix if the CNN model is capable of accurately classifying the different damage cases.

#### 2.3.1. Simulation Setup

**Figure 4:**
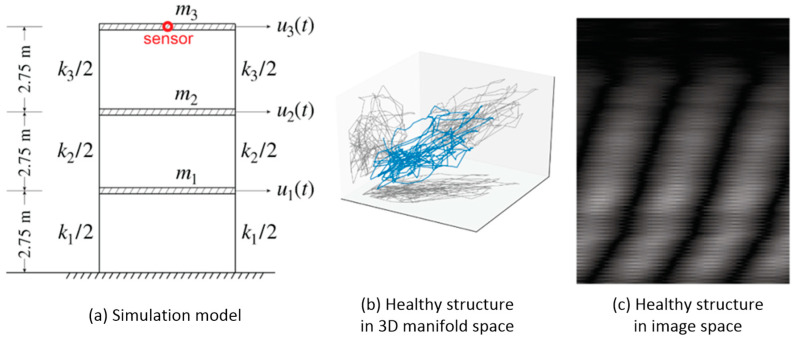
Three-story shear frame. The excitation comes from the ground, and the response of the third floor (roof) is collected. (**a**) Illustration of the three-story shear frame. (**b**) A typical attractor of the healthy structure in a three-dimensional reconstruction space. (**c**) A typical attractor of the healthy structure in the image space.

Figure 4 illustrates the simulated three-story shear frame structure and its healthy state in both the reconstruction space and the image space. Rigid floor diaphragms were considered and the masses were assumed concentrated on the diaphragms. As a result, each floor has only horizontal movement, or one DOF. The mass, stiffness, and height of each story are listed in Table 1 [31].

A Gaussian white noise signal with a length of ninety seconds was used for the ground motion excitation of the structure. The peak ground acceleration (PGA) in each ground motion event was scaled to 0.25 m/s^2^. A series of dynamic time-history analyses were carried out using the Newmark-β linear acceleration method [32] implemented in MATLAB. The time increment was set to five milliseconds in the dynamic analysis. The system output was collected on the third floor.

In this study, varying levels of structural damage were simulated through the stiffness reduction factor $R_{k}$, which denotes the remaining stiffness of a certain story. For example, an $R_{k}$ of 0.9 on the first floor indicates a reduction of stiffness by 10% on the first floor. A value of 0.9 was used for slightly damaged structures, 0.8 for moderately damaged structures, and 0.7 for a severely damaged structure. Only one story was considered as damaged ($R_{k}<1.0$) in each simulation run while the other stories had an $R_{k}$ value of 1.0. The damage combinations form the ten classes shown in Table 2. The case names are also given in the table.

**Figure 5:**
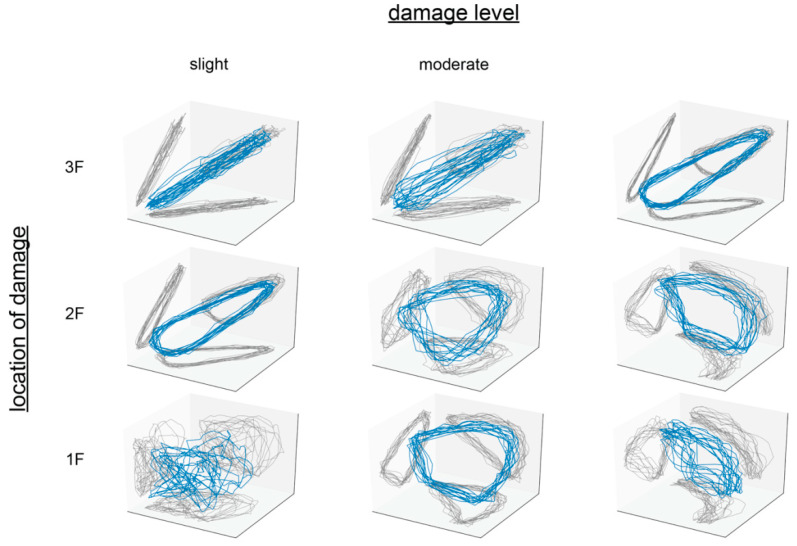
Examples of the attractor in a three-dimensional reconstruction space.

**Figure 6:**
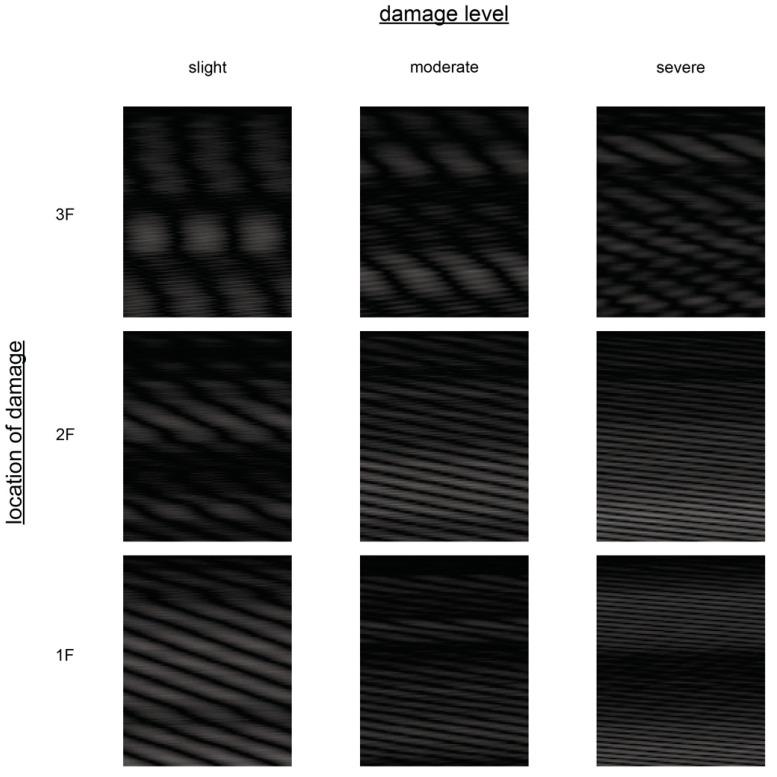
Examples of the reconstruction in the image space. These correspond to the reconstructions shown in Figure 5.

Attractors in a three-dimensional reconstruction space are depicted in Figure 5. Images converted from attractors with sample interval r=0.005 s, delay time τ=120 steps×r s=0.6 s, trajectory length T=120 steps×r s=0.6 s, and embedding dimension d=150 are presented in Figure 6.

#### 2.3.2. Convolutional Neural Network Setup

**Figure 7:**
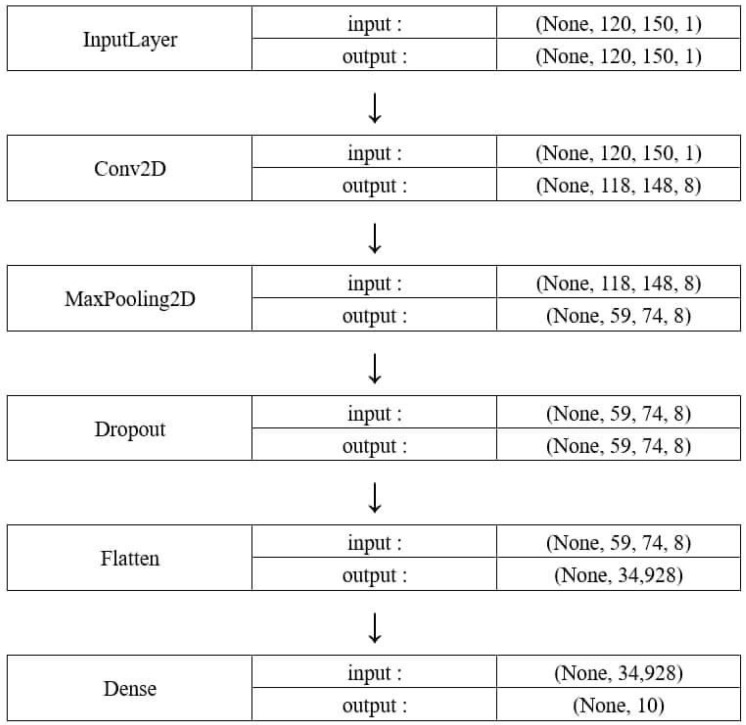
Structure of the CNN model.

The CNN model, as illustrated in Figure 7, consists of two trainable layers: the convolutional layer and the fully connected layer. The convolutional layer has eight kernels, each 3 × 3 in size. This is followed by a 2 × 2 max-pooling layer with the stride set to 2 in both dimensions, and then a dropout layer with a dropout rate of 0.5 and a flattening layer. The fully connected layer follows. Since this fully connected layer is also the output layer of the model, it consists of ten nodes to match the ten damage cases. The output of the ten nodes is fed into a softmax function to give the probability of each class. ReLU activation is used throughout the model, except for the aforementioned softmax at the output end. The ADAM optimizer is used for gradient decent, along with cross-entropy as the loss function. There are 139,796 trainable parameters in total, eighty of which belong to the convolutional kernels. The model was implemented using Python with the open-source Keras library [33], with Tensorflow as the backend [34].

## 3. Results

The displacement responses of the structure were converted into matrices with the previous parameters. As a result, the generated images have a dimension of 120×150 (width x height). A total of 2000 were generated for each of the 10 damage cases. This dataset was further divided into a training dataset and a validation dataset with 1600 images and 400 images, respectively. The set of images showcased in Figure 6 is taken from this dataset.

Three different indices are examined to quantify the results: the accuracy, recall, and Cohen’s kappa coefficient. After 20 epochs of training, both the training and validation accuracy plateaued around 99%. No apparent sign of overfitting is present, most likely due to the simplicity of the chosen CNN model.

**Figure 8:**
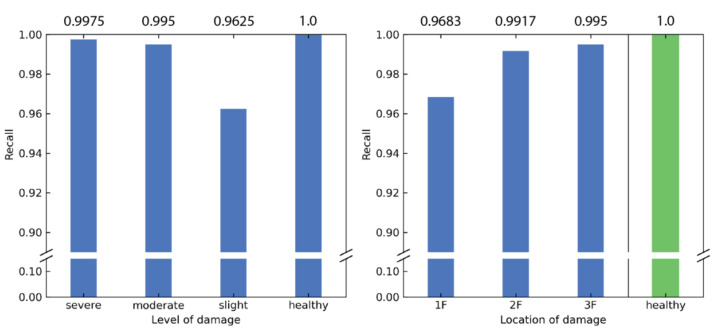
Recall calculated based on level of damage and the location of damage.

Some insights can be inferred by a close examination of the confusion matrix. To prevent sporadic results due to stochasticity, the following trends were checked against 29 different runs of the CNN training using the same CNN model with different pseudorandom seeds. Recall, also known as sensitivity, is defined as the proportion of actual positive cases that are correctly classified, given as

(4) $$\mathrm{Recall}=\frac{\mathrm{TP}}{P}=\frac{\mathrm{TP}}{\mathrm{TP}+\mathrm{FN}},$$

where TP is the number of true positives that the model correctly classified, P is the number of cases classified as positive by the model, and FN is the number of false negatives that the model classified incorrectly. It was found that the recall value was higher than 0.9 for all 10 classes. The results are summarized in Figure 8. There is a clear trend that when recall is calculated with respect to the level of damage, ease of prediction increases with increasing proximity of damage to the sensor (third floor). By contrast, severely damaged structures are easier to predict than slightly damaged structures, with the exception that healthy structures have the highest recall value. 

**Figure 9:**
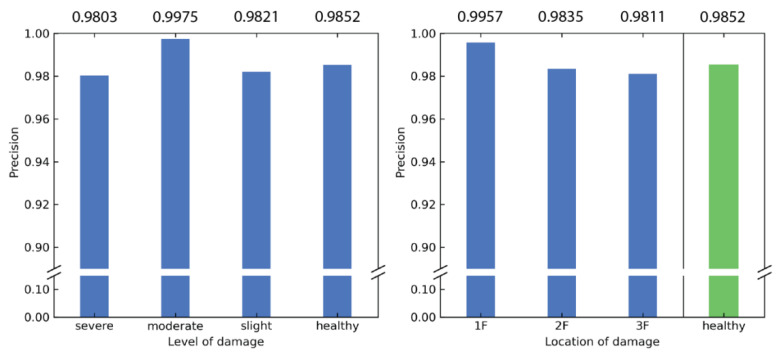
Precision calculated based on level of damage and location of damage.

The counterpart of recall is precision, which is given by

(5) $$\mathrm{Precision}=\frac{\mathrm{TP}}{\mathrm{TP}+\mathrm{FP}},$$

where TP is defined previously and FP is the number of false positives that the model incorrectly classified as positives. Similar to the recall, the precision is high for all 10 classes, with no classes having a precision less than 0.9. The results for the precision are summarized in Figure 9. It can be interpreted that the predictions are more trustworthy when the model reports damage closer to the ground floor. 

Cohen’s kappa coefficient κ is calculated as

(6) $$\kappa =\frac{p_{o}-p_{c}}{1-p_{c}},$$

where $p_{o}$ denotes the accuracy and $p_{c}$ denotes the hypothetical probability of chance agreement, which is 2000/20000=0.1 for the present demonstration. It follows that κ for this example is 0.985. With the common interpretation of Cohen’s kappa coefficient, κ=0.985 translates to almost perfect agreement with the true value. Concluding the above, the proposed conversion method does effectively embed the structural health data, in such a way that the information can be easily extracted by CNN models.

## 4. Discussion

The most efficient SHM method is to monitor the natural frequencies variation of considered structures after events. Table 3 demonstrates the natural frequencies of the three-story shear frame under healthy and different damaged scenarios, while the relative errors of natural frequencies between the healthy and damaged structures are listed in the parentheses. One can find that the more damaged scenario shows the larger frequency variation. For cases of little-damaged structures (with the reduction factor *R_k_* = 0.9) in Table 3, the relative errors of the natural frequency for the first mode are less than 3%. In most practical in situ measurements with signal noises and measurement errors, such differences are not easily identified from the measured time-history data. These results reveal the limitation of the frequency-based SHM method and the superiority of the proposed SHM method.

In order to examine whether the damping influences the structural health diagnosis of the proposed method, we consider two cases, including healthy and SE3 with a classical damping matrix **C** = diag.<200, 200, 100> kN.s/m. Each of the cases includes 2000 images. These data are divided into 1500 training dataset and 500 validation dataset. The CNN model is composed of eight convolutional layers and a fully connected layer. The 8 convolutional kernels are all in the same size of 3 × 3 but with different depth of 32, 32, 16, 16, 8, 8, 4 and 4 sequentially. Relu and softmax functions are chosen as the activation functions for the first four layers and the last four layers, respectively. The fully connected layer consists of two nodes, the healthy case and the damaged case for damage detection. The model with the highest validation loss is stored for tests. Untrained 1000 healthy cases and 1000 damaged cases are used to test the performance of the model. The derived testing accuracy of this model is 96.95%. Furthermore, its recall and precision are 95.30% and 98.55%, respectively. The evaluation index shows the ability to classify the damaged and healthy cases when damping is considered.

As mentioned in the introduction, Overbey et al. [25] reported the generalization of deterministic attractors to pullback attractors. The importance of this extension is that an exact excitation signal is no longer required. In the present example, it was demonstrated that the time delay method yields the desired and expected results with a stochastic signal as excitation, which further supports the conclusion of the former work. This generality is particularly useful when either the system in question is not suitable for giving a prescribed excitation, or the excitation that led to the gathered response is not available. In both cases, the present findings also suggest that the time delay method can be used without modification.

There are some important implications resulting from the present CNN model successfully classifying the location of damage. Most current SHM methods are unable to extract the location of damage using the time-history data of a single sensor. By successfully differentiating the location, the presence of information on the location of damage in the reconstruction attractor is proven. However, how this information should be extracted in practical usage remains unknown. This is because, in practice, the only data available are those belonging to the healthy state, and so the responses of different damage cases are unknown. Unsupervised CNN learning is therefore required. The health of the structure can only be determined by comparing the response sequence of interest to the healthy response, as commonly seen in other vibration-based damage detection schemes. Without the knowledge of other damage cases, detecting the location of damage would be even more challenging.

Some properties of the attractor matrix are discussed here. Apart from the evident effect of T and d on the dimensions of the matrix, the combination of T, r, and τ affects the uniqueness of each element. More specifically, when T×r is larger than τ, values in the last few columns reappear in the first few columns, shifted down by one row. This has some practical consequences. For the typical case where a lengthy time-history response is collected at sample rate r, the delay time τ and the length T of the trajectory must be chosen carefully if the user is to maximize the amount of information in the matrix, i.e., to avoid repeating values. It should be noted that in the special case where the sample rate r is equal to the delay time τ, the attractor matrix becomes a Hankel matrix:

(7) $$\left[\begin{array}{ccccc} x\left(t_{0}\right) & x\left(t_{0}+\tau \right) & x\left(t_{0}+2\tau \right) & \cdots & x\left(t_{0}+\left(T-1\right)\tau \right)\\ x\left(t_{0}+\tau \right) & x\left(t_{0}+2\tau \right) & x\left(t_{0}+3\tau \right) & \cdots & x\left(t_{0}+T\tau \right)\\ \vdots & \vdots & \vdots & \ddots & \vdots \\ x\left(t_{0}+\left(d-1\right)\tau \right) & x\left(t_{0}+d\tau \right) & x\left(t_{0}+\left(d+1\right)\tau \right) & \cdots & x\left(t_{0}+\left(d+T\right)\tau \right) \end{array}\right],$$

Although the attractor matrix is formulated with the time delay vectors as row vectors, other interpretations of the matrix can be made. The CNN can therefore see more than just the attractor that formulates the matrix. As an example, the transpose of the matrix is shown to be a second attractor in the same format as in the proposed method. The original matrix M is constructed with sample rate r, delay time τ, trajectory length T, and embedding dimension d. The transpose of M, denoted by $M^{T}$, is

(8) $$\left[\begin{array}{ccccc} x\left(t_{0}\right) & x\left(t_{0}+\tau \right) & x\left(t_{0}+2\tau \right) & \cdots & x\left(t_{0}+\left(d-1\right)\tau \right)\\ x\left(t_{0}+r\right) & x\left(t_{0}+\tau +r\right) & x\left(t_{0}+2\tau +r\right) & \cdots & x\left(t_{0}+\left(d-1\right)\tau +r\right)\\ \vdots & \vdots & \vdots & \ddots & \vdots \\ x\left(t_{0}+\left(T-1\right)r\right) & x\left(t_{0}+\tau +\left(T-1\right)r\right) & x\left(t_{0}+2\tau +\left(T-1\right)r\right) & \cdots & x\left(t_{0}+\left(d-1\right)\tau +\left(T-1\right)r\right) \end{array}\right],$$

**Figure 10:**
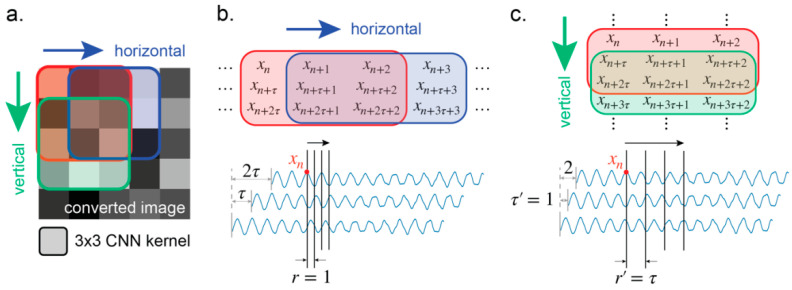
(**a**) Two of the possible interpretations by the CNN of the image: horizontally and vertically. (**b**) With the horizontal interpretation, the parameters of the reconstruction are the same as in the proposed method. (**c**) The vertical interpretation is equivalent to using an alternative sample rate of τ and delay time of 1.

The parameters of reconstruction for $M^{T}$ are further denoted with the superscript T. By comparison, it is found that the transposed sample rate $r^{T}$ is τ, the transposed delay time $\tau^{T}$ is r, the transposed trajectory length $T^{T}$ is d, and the transposed embedding dimension $d^{T}$ equals T. Conceptually, if the matrix M is analyzed through a horizontally sliding window, the original trajectory that is constructed by the set of un-superscripted parameters {r,τ,T,d} can be seen. By contrast, the trajectory constructed by the set of superscripted parameters $\left\{r^{T},\tau^{T},T^{T},d^{T}\right\}$ is observed if the window is slid vertically (Figure 10).

## 5. Conclusions

Machine learning techniques in image classification, specifically CNN, have been applied fruitfully in multiple disciplines. CNNs are intrinsically high-dimensional and suitable for handling high-dimensional data, making possible the exclusion of dimensional reduction in data processing. To use this advantage, a method is proposed to convert high-dimensional attractors into matrices/images readily available for CNN models. In particular, the considered sequence of observation is obtained from a single sensor on a multi-DOF structure. It is then desirable to construct a representative attractor of the underlying dynamical system from such a sequence. This is achieved using Takens’ embedding theorem; time-delayed versions of this output signal can be gathered to form an attractor with the number of DOF matching that of the original system. By the definition of DOF, the structure can then be fully described in this embedding space. The time-series data are first coupled with delayed copies of itself to form an attractor, which is then presented in matrix form. A simulation of a two-dimensional three-story shear frame was conducted as a numerical validation of the proposed method. The results show that a simple CNN can discriminate the 10 different damage cases at an accuracy of 99%. It is therefore concluded that the conversion has successfully embedded the information of the attractor into the image/matrix and is suitable for extraction with CNN computation. We anticipate that the proposed attractor identification method could be used for a wide range of engineering applications with high dimensional dynamical systems.

## Figures and Tables

**Table 1:** Parameters of the three-story shear frame.

Story	Mass (kg)	Stiffness (kN/m)	Story Height (m)
1	*m*_1_ = 65.00 × 10^3^	*k*_1_ = 76,363.6	*h*_1_ = 2.75
2	*m*_2_ = 59.76 × 10^3^	*k*_2_ = 61,090.9	*h*_2_ = 2.75
3	*m*_3_ = 49.54 × 10^3^	*k*_3_ = 61,090.9	*h*_3_ = 2.75

**Table 2:** Ten simulation setups comprising the ten classes.

Case Name	Stiffness Reduction Factor *R_k_*		
	First Floor	Second Floor	Third Floor
Healthy	1.0	1.0	1.0
SL1	0.9	1.0	1.0
MO1	0.8	1.0	1.0
SE1	0.7	1.0	1.0
SL2	1.0	0.9	1.0
MO2	1.0	0.8	1.0
SE2	1.0	0.7	1.0
SL3	1.0	1.0	0.9
MO3	1.0	1.0	0.8
SE3	1.0	1.0	0.7

**Table 3:** Natural frequencies of ten simulation setups.

Case Name	Natural Frequencies (Hz)		
	First Mode	Second Mode	Third Mode
Healthy	2.51	6.67	9.26
SL1	2.44 (2.72%)	6.54 (1.96%)	9.21 (0.53%)
MO1	2.36 (5.86%)	6.41 (4.01%)	9.17 (1.02%)
SE1	2.27 (9.54%)	6.26 (6.14%)	9.12 (1.46%)
SL2	2.46 (2.13%)	6.63 (0.65%)	9.13 (2.43%)
MO2	2.39 (4.63%)	6.58 (1.46%)	8.81 (4.83%)
SE2	2.32 (7.62%)	6.51 (2.46%)	8.60 (7.15%)
SL3	2.50 (0.57%)	6.49 (2.73%)	9.08 (1.91%)
MO3	2.48 (1.28%)	6.27 (6.00%)	8.93 (3.61%)
SE3	2.46 (2.21%)	6.02 (9.84%)	8.79 (5.11%)

## Data Availability

The data presented in this study are available on request from the corresponding author.
